# Optimization of Intraoperative Near-Infrared Fluorescence Mapping with Indocyanine Green for Sentinel Lymph Node Detection in Cervical and Endometrial Cancer

**DOI:** 10.3390/pharmaceutics18020211

**Published:** 2026-02-06

**Authors:** Kanamat Efendiev, Maria Meshkova, Polina Alekseeva, Andrei Udeneev, Arkadii Moskalev, Maxim Loshchenov, Heda Maltsagova, Svetlana Mukhtarulina, Andrey Kaprin, Victor Loschenov

**Affiliations:** 1 Prokhorov General Physics Institute of the Russian Academy of Sciences, Moscow 119991, Russia; 2 Department of Laser Micro-, Nano-, and Biotechnology, Institute of Engineering Physics for Biomedicine, National Research Nuclear University “MEPhI”, Moscow 115409, Russia; 3 Institute of Mathematics and Natural Sciences, Kabardino-Balkarian State University, Nalchik 360004, Russia; 4 Lopatkin Research Institute of Urology and Interventional Radiology, Moscow 105425, Russia; 5 National Medical Research Radiological Center of the Ministry of Health of Russian Federation, Obninsk 249036, Russia

**Keywords:** indocyanine green, fluorescence mapping, fluorescence imaging, near-infrared imaging, sentinel lymph node, cervical cancer, endometrial cancer, gynecologic oncology

## Abstract

**Background/Objectives:** Lymph node dissection during surgeries for cervical and endometrial cancer is associated with significant complications and morbidity. Sentinel lymph nodes (SLNs) mapping using indocyanine green (ICG) has become a promising method for reducing surgical invasiveness and improving patient outcomes. However, the optimal protocol for intraoperative fluorescence mapping of SLNs using ICG, especially regarding the timing of imaging after injection, remains to be fully optimized. This study aimed to evaluate the efficacy of real-time near-infrared (NIR) fluorescence SLN mapping at various time intervals and to investigate the photophysical properties of ICG in human lymph to establish a correlation between fluorescence signals and dye concentration. **Methods:** A prospective study included 20 patients with cervical and endometrial cancer undergoing laparoscopic or laparotomic surgery. Interstitial ICG injection was administered into the cervical stroma. SLN mapping was conducted using the novel VENERA-green endoscopic system (λ_exc_ = 800 nm, registration of fluorescence in the range of 830–1000 nm). Spectral fluorescence analysis (λ_exc_ = 650 nm) was conducted on SLNs and optical phantoms containing human lymph with ICG concentrations from 0 to 40 mg/L. The method made it possible to evaluate ICG absorption/emission properties, as well as to quantify concentration-dependent effects. **Results:** SLNs were successfully detected in all patients. The average detection time was 15 min with a range of 10 to 25 min. Fluorescence intensity of SLNs was significantly higher 10–15 min after ICG injection compared to 20–25 min. Spectral analysis indicated an absorption peak at 804 nm and an emission peak in the 835–855 nm range for ICG in human lymph. A concentration-dependent redshift of the fluorescence peak was observed and accurately modeled using a logarithmic function (R^2^ = 0.99), which allows for the estimation of ICG concentration in tissue. The bilateral detection rate was 77% for laparoscopy and 100% for laparotomy. Metastases were histologically confirmed in only 2.8% (1/36) of the detected SLNs. **Conclusions:** Intraoperative NIR fluorescence imaging using ICG is a highly sensitive method for real-time SLN mapping in gynecologic oncology. The optimal detection period is 10 to 15 min after cervical injection to achieve maximum ICG fluorescence intensity, compared to 20 to 25 min. The concentration-dependent fluorescence and absorption properties of ICG in lymph provide the basis for the development of quantitative intraoperative monitoring methods that could improve the accuracy of sentinel lymph node biopsy.

## 1. Introduction

It is well-established that lymph node dissection is associated with serious postoperative complications, leading to a significant reduction in patients’ quality of life. The incidence of lymphocyst can be as high as 47.6–49.4%, with symptomatic lymphocysts developing in 5.2–5.6% of cases [1,2]. Lymphedema is reported in 20% of cases, and lower urinary tract dysfunction occurs in 10–15% [3].

Furthermore, in patients with morbid obesity, endometriosis, or adhesions, lymph node dissection presents technical challenges and, consequently, a high rate of intraoperative complications [4]. Currently, sentinel lymph node (SLN) mapping is a promising method that can avoid excessive surgical tissue trauma.

The most commonly used methods for intraoperative SLN detection include:

- Fluorescence-guided navigation with interstitial administration of indocyanine green (ICG) [5];
- Visual assessment of the distribution of the blue dye in tissues [6];
- Detection of radioactivity from radioactive colloids in tissues [7];
- Imaging of magnetic nanoparticles in tissues [8].

The use of blue dyes for SLN mapping has several drawbacks. A study [9] reported SLN detection rates of 89% and 100% for methylene blue (MB) and ICG, respectively. According to research [10], the bilateral detection rate for SLN using MB in combination with ^99m^Tc is significantly lower than with ICG, at 73.5% and 90.3%, respectively. The administration of radioactive colloids exposes patients to ionizing radiation, and the imaging modality suffers from low spatial and temporal resolution [11]. A study [12] reported an overall SLN detection rate of 95% for the radioisotope ^99m^Tc compared to 98% for ICG. However, when assessing the bilateral detection rate, the use of the ^99m^Tc radioisotope demonstrated lower identification rates compared to ICG—63% and 85%, respectively. The magnetic nanoparticle technique for SLN detection is still relatively limited in practical application and remains in the stage of clinical investigations [8,13].

SLN mapping using ICG demonstrated higher detection rates compared to other techniques. This method offers high sensitivity and enables real-time fluorescence imaging of LNs [14] with minimal contribution from tissue autofluorescence in the recorded signal (~700–900 nm). It also avoids exposure to ionizing radiation [15]. Furthermore, ICG enhances the likelihood of bilateral SLN detection, thereby reducing the number of unnecessary lymphadenectomies when LNs are not visualized.

ICG-based SLN mapping is recommended for patients diagnosed with endometrial cancer (EC) and cervical cancer (CC), as reflected in both Russian and international clinical guidelines [16,17,18,19,20]. It is essential to emphasize that the application of ICG in modern medicine, and specifically in gynecologic oncology, extends far beyond simple SLN mapping. Due to its excellent safety profile, rapid systemic clearance, and unique optical properties in the near-infrared (NIR) spectrum, ICG has become the “gold standard” in fluorescence-guided surgery (FGS).

In contemporary surgical oncology, ICG serves as a pivotal tool for SLN identification across a broad spectrum of malignancies, including breast cancer [21], melanoma [22], and gynecological cancers [23]. Research confirms that ICG-based imaging provides detection rates and sensitivity comparable to traditional radiocolloid methods while eliminating radiation exposure for both patients and surgical personnel [24]. Beyond lymphology, ICG-based NIR fluorescence is critical for the intraoperative assessment of tissue perfusion and microvascular viability [25]. In colorectal and esophageal surgery, real-time visualization of anastomotic sites significantly reduces the risk of leakage and prevents postoperative complications [26,27]. In hepatobiliary surgery, fluorescent cholangiography provides precise visualization of the biliary anatomy, minimizing the risk of iatrogenic injury during cholecystectomy [28].

Recent advancements have further expanded the clinical applications of ICG into specialized fields, such as urology, aiding in renal vasculature visualization during partial nephrectomy [29] and pediatric surgery [30]. Furthermore, there is growing interest in nerve visualization, tissue flap viability assessment during reconstructive procedures, and innovative approaches in phototheranostics [23,31,32].

However, despite such widespread clinical adoption, the vast majority of existing FGS protocols are limited to qualitative (visual) assessment of the fluorescence signal. Quantitative monitoring of ICG accumulation dynamics and the analysis of its spectral behavior as a function of concentration in SLNs remain largely unexplored. The present study focuses primarily on the transition from subjective visualization to objective quantitative analysis of ICG spectral characteristics in SLNs.

**Figure 1:**
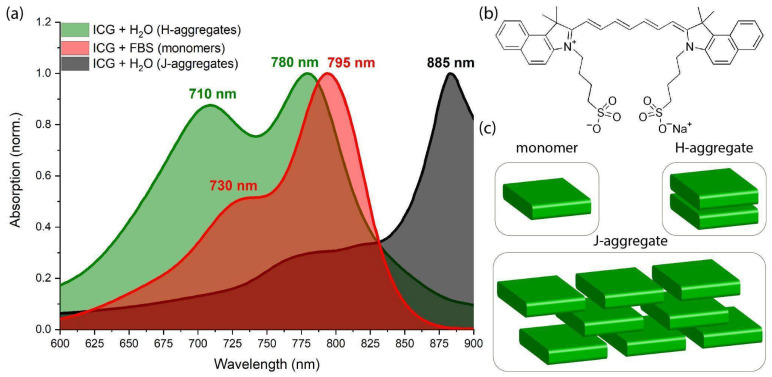
(**a**) Absorption spectra of ICG solutions. (**b**) Chemical structure of ICG. (**c**) Schematic illustration of the formation of H-aggregates and J-aggregates. The spectra in the figure are normalized to the maximum absorbance value. The absorption spectra of ICG dissolved in water for injection are represented in green and gray, while the spectrum of ICG dissolved in FBS is shown in red. The ICG concentration is 50 mg/L in all cases.

ICG is a water-soluble tricarbocyanine dye. Its absorption and fluorescence properties are highly dependent on the dye’s concentration and the solvent used. ICG absorbs light in the 600–900 nm range (with a peak in blood plasma at ~780 nm) and fluoresces from 750 to 950 nm. In an aqueous solution, ICG primarily exists as monomers and H-type dimers (H-aggregates), characterized by two absorption peaks at ~780 nm and ~710 nm, respectively (Figure 1a). In blood or lymph, ICG molecules can bind to proteins, which stabilizes and maintains the distance between molecules, allowing ICG to exist predominantly in its monomeric form. The absorption peaks of H-aggregates and monomers of ICG in fetal bovine serum (FBS) are redshifted, with maxima at ~730 nm and ~795 nm, respectively. Over time, in an aqueous solution, ICG dimers and monomers can transform into J-aggregates with an absorption peak in the region of 885 nm (Figure 1a,c).

A limitation of ICG application, besides individual drug intolerance [33], is the strong dependence of its absorption and fluorescence properties on the solvent environment and the dye’s own concentration [34]. In aqueous solutions, the absorption of ICG at high concentrations shifts to the ~700 nm region, indicating the presence of H-aggregates [35], while the monomeric peak at ~780–805 nm decreases [36]. The aggregation process of ICG molecules is also influenced by the microenvironment in biological tissues. This can cause changes in fluorescence intensity and a shift in the fluorescence peak [37]. Blood serum can dissolve ICG at high concentrations, potentially leading to a linear increase in fluorescence intensity [36]. Thus, the fluorescence intensity of ICG in vivo can vary depending on fluctuations in the concentration of plasma proteins and lipoproteins to which ICG binds [38], as well as on the time interval between ICG administration and the initiation of fluorescence navigation.

For SLN detection in EC and CC, interstitial injection of ICG is used, creating multiple “ICG depots”. In such cases, the fluorescence signals from nearby lymphatic vessels and SLNs may be detected with a delay due to the high ICG concentration (concentration quenching), and the fluorescence intensity becomes brighter after dilution by proteins over several minutes [39].

One of the main goals of this study is to develop a method for the quantitative intraoperative monitoring of ICG accumulation. In contrast to traditional approaches, which are limited to the qualitative visualization of “signal presence,” our research focuses on analyzing ICG spectral changes directly within the human lymph environment. This approach enables not only the detection of SLNs but also an objective, real-time assessment of ICG concentration dynamics, which is crucial for optimizing the surgeon’s “diagnostic window” and enhancing mapping accuracy.

This study evaluated the effectiveness of fluorescence-based SLN mapping during laparoscopic and laparotomic surgeries for patients with EC and CC. The evaluation took place at various time intervals after the interstitial injection of ICG into the cervical stroma. In vivo video- and spectral fluorescence diagnostics were conducted on the identified SLNs. Additionally, the study investigated the absorption and fluorescence properties of ICG dissolved in human lymph at various concentrations. A comparison of the fluorescence properties of SLNs with phantom samples was performed to assess the distribution of ICG concentration at the time of visualization and biopsy. For the first time, this research explored the in vivo dynamics of ICG accumulation in SLNs after its injection into cervical tissue. The study also presented a novel endoscopic video fluorescence system for effective real-time SLN mapping by excitation of ICG fluorescence in the NIR range.

## 2. Materials and Methods

### 2.1. Indocyanine Green

For fluorescence navigation, Indocyanine Green^®^ (FERMENT LLC., Moscow, Russia), a commercially available agent, was used. This product is supplied as a brownish-green powder or a porous mass with a violet tint. To prevent exposure to sunlight and photobleaching, the ICG was stored in a light-protected container at 2–8 °C.

**Figure 2:**
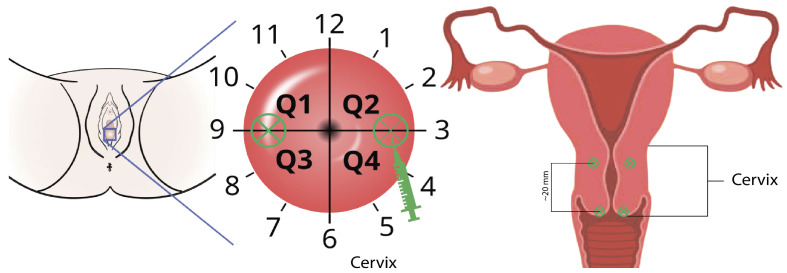
Cervix quadrants and directions (ICG injection zones are highlighted with a green marker).

Immediately before surgery, 25 mg of ICG powder was reconstituted in 20 mL of sterile water for injection. The total dose for interstitial injection was 5 mg (total volume of 4 mL). The ICG was injected into the cervical stroma at the 3 and 9 o’clock positions. The administration included four injections in total: two superficial (~2 mm, 1 mL each) and two deep (~20 mm, 1 mL each) (Figure 2).

This two-tiered injection method was employed to facilitate SLN detection. Following the injection, an atraumatic uterine manipulator was typically placed for laparoscopic procedures.

### 2.2. Fluorescence Endoscopic Video System

**Figure 3:**
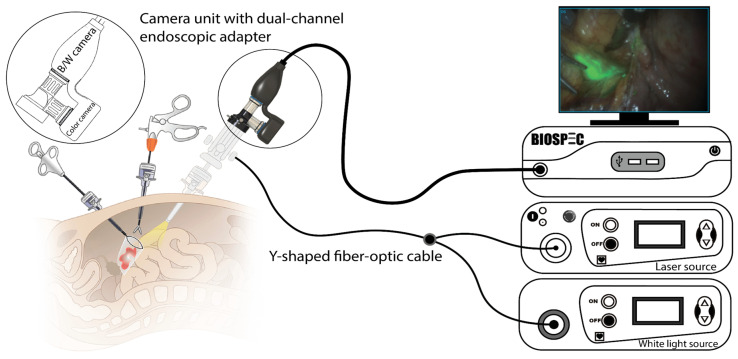
Schematic diagram of video fluorescence imaging using the VENERA-green endoscopic video system.

In the study, the VENERA-green endoscopic video system (BIOSPEC Ltd., Moscow, Russia) was used for intraoperative NIR fluorescence navigation. This system consisted of a white light source, a semiconductor NIR laser, a Y-shaped fiber-optic cable, and a camera module with a dual-channel endoscopic adapter. The camera module is equipped with highly sensitive monochrome (B/W) and color (RGB) cameras and can be coupled with an endoscope or laparoscope (Figure 3).

For fluorescence imaging, the system acquires images in three modes: color (navigation), monochrome (fluorescence), and an overlay (merged) mode. ICG fluorescence is excited at a wavelength (λ_exc_) of 800 ± 5 nm with a laser power density at the surface of 20–30 mW/cm^2^ and is detected in the wavelength range of 830–1000 nm. The examples of intraoperative NIR fluorescence imaging of SLN using ICG in overlay mode are presented in Supplementary Materials (Videos S1 and S2).

In the overlay mode, dedicated software synchronizes the operation of the monochrome and color cameras. It displays a real-time color image overlaid with the corresponding monochrome fluorescence image. Fluorescence areas are displayed in a “green color” pseudocolor, which allows for visual determination of SLN boundaries and calculation of the fluorescence contrast coefficient within a selected region of interest. Fluorescence-based SLN mapping using the endoscopic video system was performed during both laparoscopic and laparotomic surgeries.

### 2.3. Spectral Fluorescence Diagnostics

Spectral fluorescence studies of SLNs and optical phantoms were performed using the LESA-01-BIOSPEC spectroscopic system (BIOSPEC Ltd., Moscow, Russia) [40]. An optical filter was placed in front of the spectrometer to enable the registration of both diffusely backscattered laser light and NIR fluorescence within a single dynamic range. This was achieved by attenuating radiation in the 620–680 nm wavelength range and transmitting light in the 710–1000 nm spectral range.

**Figure 4:**
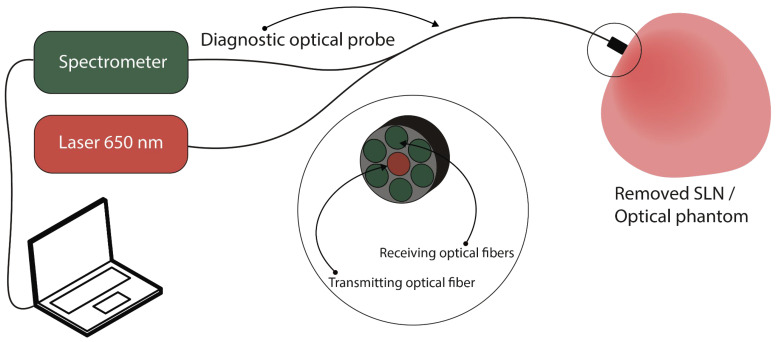
Schematic diagram of spectral fluorescence diagnostics using the LESA-01-BIOSPEC spectroscopic system.

The delivery of the excitation laser radiation and the collection of fluorescence were accomplished using a diagnostic optical probe. The diagnostic optical probe consisted of 6 receiving optical fibers arranged concentrically around a single central transmitting optical fiber dedicated to delivering laser light to the surface of the tissue (Figure 4).

The optical fibers had a diameter of 250 µm and a numerical aperture of NA = 0.22.

ICG fluorescence was excited by a laser with a wavelength of 650 ± 5 nm and an output power at the light guide of 10–12 mW. The exposure time for recording spectral data varied from 20 to 100 ms. At each time point, 3–5 spectra were recorded and subsequently averaged.

### 2.4. Histopathology

The identified SLNs were extracted from the abdominal cavity during laparoscopic surgery using a sealed endoscopic retrieval bag (EndoBag) and sent for urgent intraoperative morphological examination to determine the presence or absence of metastases. The SLNs were sectioned into 2–4 parallel slices and subsequently stained with hematoxylin and eosin for analysis. Following the intraoperative assessment, all examined SLNs were sent for planned, comprehensive histological examination.

### 2.5. Optical Phantoms

Immediately before the spectral investigation of the absorption and fluorescent properties of ICG in SLNs, optical phantoms were prepared. These phantoms were based on human lymphatic fluid, with a volume of 500 µL, and contained ICG^®^ (Firm FERMENT LLC., Moscow, Russia) at concentrations of 0, 2, 5, 10, 20, 30, and 40 mg/L. The human lymph was provided by the Lopatkin Research Institute of Urology and Interventional Radiology. The optical phantoms were prepared immediately before conducting the spectral fluorescence studies.

Spectral fluorescence diagnostics of the optical phantoms were performed using the LESA-01-BIOSPEC spectrometer (BIOSPEC Ltd., Moscow, Russia). Absorption spectra were recorded in the 600–900 nm range using a Hitachi U3900 spectrophotometer (Hitachi Ltd., Tokyo, Japan) in quartz cuvettes with an optical path length of 1 cm.

### 2.6. Statistical Analysis

All data are presented as mean ± standard deviation (SD). The Shapiro–Wilk test was used to assess the normality of data distribution. The significance of differences was tested using Student’s *t*-test. A *p*-value of less than 0.05 was considered statistically significant for all tests.

## 3. Results

Immediately following the injection of ICG into the cervical stroma, NIR fluorescence mapping of SLNs was performed for all 20 patients undergoing both laparoscopic (85%) and laparotomic (15%) procedures. In each case, the primary objective was to identify and biopsy the SLNs.

**Figure 5:**
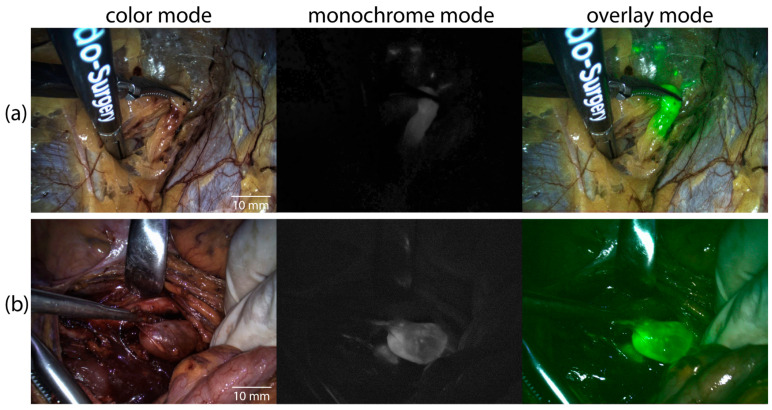
NIR fluorescence mapping of ICG-accumulating SLNs using the VENERA-green endoscopic video system, demonstrating the three imaging modes (color, monochrome, and overlay mode): (**a**) Laparoscopic surgery; (**b**) Laparotomic surgery.

In addition to providing intraoperative navigation for surgical maneuvers, the VENERA-green endoscopic video system enabled real-time NIR mapping of lymph nodes (LNs), including SLNs, with precise delineation of their boundaries. Representative examples of video fluorescence imaging results of ICG-accumulating SLNs during laparoscopic and laparotomic surgeries are presented in Figure 5.

**Figure 6:**
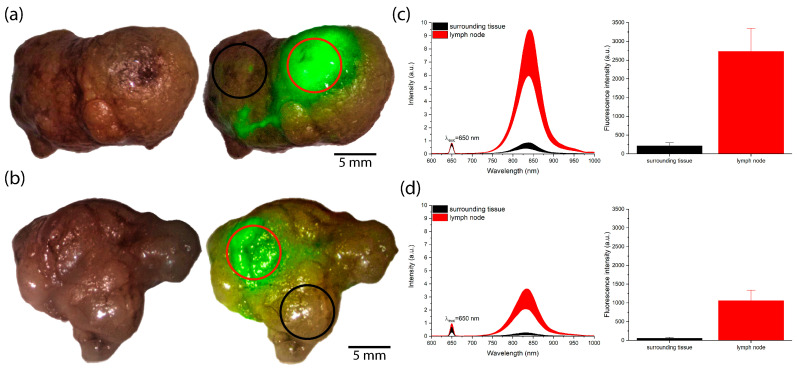
Results of video and spectral fluorescence diagnostics of SLNs: (**a**,**b**) Images in color and overlay modes (red and black markers indicate regions where spectral fluorescence analysis was performed); (**a**) 10–15 min after ICG injection; (**b**) 20–25 min after ICG injection; (**c**,**d**) Results of spectral fluorescence analysis of selected regions and distribution of integral fluorescence intensity of ICG in the 750–950 nm range; (**c**) 10–15 min after ICG injection; (**d**) 20–25 min after ICG injection.

Video fluorescence imaging was performed in three modes: color, monochrome, and overlay. In the overlay mode, areas with intense fluorescence were highlighted in green. Examples of intraoperative video fluorescence imaging of SLNs in the overlay mode are provided in the Supplementary Materials (Videos S1 and S2). Additionally, following the biopsy of the SLNs, video fluorescence imaging (Figure 6a,b) and spectral fluorescence diagnostics (Figure 6c,d) were performed on the areas of intense ICG fluorescence and the surrounding tissues.

The identified SLNs were subsequently sent for histological examination to confirm the presence or absence of metastases. The mean time for SLN detection after ICG injection into the cervix was 15 min. The fluorescence intensity of the SLNs 10–15 min after injection was higher (Figure 6c) than that observed 20–25 min after ICG administration (Figure 6d). The fluorescence peaks of ICG also differed between the two time points. At 10–15 min, the fluorescence peak was in the range of 838–842 nm, with a mean maximum (λ_max_) of 840 nm. At 20–25 min, the fluorescence peak shifted to the range of 832–838 nm, with a mean λ_max_ = 835 nm.

In each case, the detected SLNs were sent for urgent intraoperative histological examination to confirm the presence or absence of metastases. Metastases in the SLNs visualized by ICG navigation were detected in only 1 out of the 20 patients (a patient diagnosed with CC). Furthermore, micrometastases were found in only one of the two identified and removed SLNs. Table 1 shows detailed results of the biopsy status for all laparoscopic (n = 17) and laparotomic (n = 3) procedures.

$$\text{Sensitivity}\;\text{of}\;\text{SLNs}\;\text{bilateral}\;\text{detection}\;=\frac{\text{number}\;\text{of}\;\text{successful}\;\text{attempts}\;\text{to}\;\text{detect}\;\text{SLNs}\;\text{on}\;\text{the}\;\text{right}\;\text{and}\;\text{left}}{\text{number}\;\text{of}\;\text{attempts}\;\text{to}\;\text{detect}\;\text{SLNs}}\times 100\%$$

The SLN detection time ranged from 10 to 25 min. In the majority of cases (75%, n = 15), ICG-based detection of SLNs was possible within 10–15 min after the injection of the ICG solution into the cervical tissue. The total operative time for all patients was 120 ± 64 min. The duration of laparotomic procedures was 220 ± 51 min with a blood loss of 100 ± 60 mL, while laparoscopic surgeries lasted 120 ± 61 min with a mean blood loss of 100 ± 58 mL. Table 2 shows the clinical parameters of the SLNs detection in all patients.

The observed variations in detection time may reflect individual differences in lymphatic drainage kinetics and anatomical characteristics. These results underscore the importance of maintaining continuous intraoperative fluorescence monitoring throughout this critical time window to ensure comprehensive SLN mapping.

**Figure 7:**
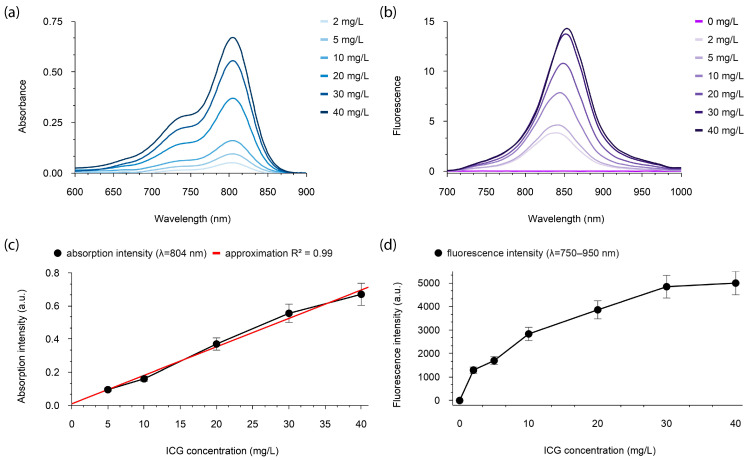
Absorption and fluorescent properties of optical phantoms based on human lymph containing ICG at concentrations of 2, 5, 10, 20, 30, and 40 mg/L: (**a**) Absorption spectra in the 600–900 nm range; (**b**) Fluorescence spectra at λ_exc_ = 650 nm; (**c**) Absorption intensity distribution of ICG at λ = 804 nm; (**d**) Distribution of integral fluorescence intensity of ICG in the 750–950 nm range.

To evaluate the absorption and fluorescent properties of ICG localized in lymph nodes, optical phantoms based on human lymph containing ICG were prepared. The optical phantoms were prepared immediately before the studies, as ICG aggregation can occur over time [41]. The absorption properties of ICG were investigated in the 600–900 nm range, and the fluorescent properties of the optical phantoms were studied in the 710–1000 nm range (Figure 7).

The absorption peak of ICG dissolved in human lymph was observed at a wavelength of 804 nm, with a shorter-wavelength “shoulder” near 740 nm (Figure 7a), while the fluorescence peak was in the range of 835–855 nm (Figure 7b). The absorption intensity increased linearly with increasing ICG concentration (Figure 7c). In contrast, the fluorescence intensity of ICG exhibited a non-linear dependence on concentration (Figure 7d).

**Figure 8:**
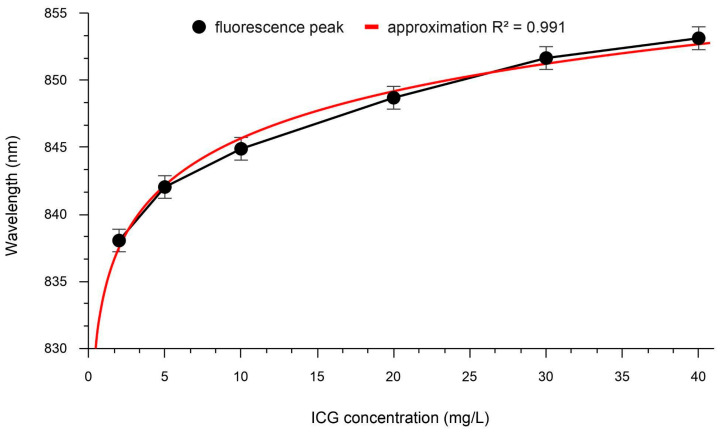
Dependence of the ICG fluorescence peak wavelength on its concentration in lymph.

Due to the significant overlap of the absorption and fluorescence spectra of ICG in the range of ~750 to ~825 nm, concentration quenching of fluorescence can be observed at high concentrations. With increasing ICG concentration, a non-linear redshift of the fluorescence peak was detected (Figure 8).

The dependence of the red shift in the ICG fluorescence peak on the ICG concentration is well approximated by a logarithmic function:

Fluorescence peak = *a* + b*ln(*C_ICG_*),

where *C_ICG_* is the ICG concentration, and *a* and *b* are proportionality coefficients, with a reliability of *R*^2^ = 0.99. This feature could potentially be used for intraoperative monitoring of ICG distribution in tissues in the future.

Based on this approximation, the ICG concentration in lymph nodes detected 10–15 min after administration could range from 2.21 to 4.87 mg/L, while in SLNs detected 20–25 min after administration, it could range from 0.68 to 2.21 mg/L. At high concentrations, ICG may form aggregates [35], and the effect of “concentration quenching” may be observed [36].

## 4. Discussion

SLN metastasis is a primary adverse prognostic factor in malignant neoplasms of the female reproductive system [41]. The frequency of SLN metastasis directly correlates with the stage of the tumor process in CC. For instance, in CC stages IB1-IB2, the frequency of SLN metastasis is 7.5%, while in stages IIA1-IIB, it reaches 36.7%. Meanwhile, the rate of lymphogenic metastasis to the para-aortic lymph nodes is 3.8% [42].

The presence of metastatic involvement in lymph nodes indicates a high risk of disease progression and serves as an indication for prescribing chemoradiotherapy [16]. In EC stages IA-IB, the frequency of SLN metastasis is 0.8% [43]. Targeted assessment of lymph node status in gynecologic malignancies is a cornerstone in determining further management strategies.

NIR fluorescence imaging technology represents a promising method with high sensitivity, enabling the avoidance of radiopharmaceuticals for SLN mapping and thereby minimizing radiation exposure for the patient. Currently, ICG is the most commonly used agent for NIR mapping of the lymphatic and circulatory systems. It is one of the least toxic dyes, with minimal interference from tissue autofluorescence (~500–700 nm) in the recorded infrared fluorescence signal. The ICG is approved by both the Food and Drug Administration (FDA) and the European Medicines Agency (EMA).

The results of the multicenter, phase 3, randomized FILM trial, which evaluated the safety and efficacy of interstitial ICG injection into the cervix, are significant to highlight [44]. A total of 176 patients diagnosed with stage I EC or CC were enrolled in the study. In the first group of 87 patients, an injection of blue dye (1% isosulfan blue) was administered into the cervix. Sentinel lymph node (SLN) identification in this group was then performed using ICG fluorescence imaging. In the second group of 89 patients, an ICG injection was administered into the cervix. Subsequently, SLN detection in this group was performed using isosulfan blue. SLNs were identified in 159 patients (98%) with ICG and in 124 patients (76%) with blue dye. The study authors concluded that ICG is more effective than blue dye for node identification. There were no reports of allergic reactions or side effects related to either the blue dye or ICG. Based on the results of this study, the application for interstitial ICG injection combined with NIR imaging for SLN mapping was approved by the FDA and has been integrated into clinical practice.

According to FDA recommendations, the standard injection points are at the 3 and 9 o’clock positions on the conventional clock face. The recommended concentration is 1.25 mg/mL, with a maximum interstitial injection dose of 5 mg (total volume of 4 mL). The injection is performed immediately before uterine manipulator placement, following coagulation of the utero-tubal junctions. Under sterile conditions, ICG is injected into the cervical stroma at the 3 and 9 o’clock positions using a syringe: first superficially (~2 mm), and then deeper (~20 mm) into the cervical stroma.

NCCN guidelines also describe alternative injection points, such as the 2, 5, 7, and 9 o’clock or 3, 6, 9, and 12 o’clock positions [17]. Some studies report subserosal/myometrial injection during laparoscopy or subendometrial injection during hysteroscopy [18]. However, these methods are technically challenging and have not been widely adopted in routine practice.

Incorrect injection technique, cervical tissue fibrosis, or disruption of lymphatic architecture may hinder SLN mapping. According to National Comprehensive Cancer Network (NCCN) guidelines, ipsilateral lymphadenectomy should be performed if no SLN is visualized [17,18].

The guidelines from the European Society of Gynaecological Oncology (ESGO), the European Society for Radiotherapy and Oncology (ESTRO), and the European Society of Pathology (ESP) state that in cases where no SLN is detected, a repeat injection of the tracer is permissible. Similarly, if SLNs are detected unilaterally, a repeat injection into the cervix is also acceptable. A study [45] demonstrated that a repeat cervical injection increased the rate of bilateral SLN detection from 78% to 94%. In our study, the rate of unilateral detection was 23% during laparoscopic procedures, whereas bilateral SLNs were identified in all cases of laparotomic surgeries. In cases of unilateral detection, a repeat ICG injection into the cervix was not performed.

The experience and high qualification of the operating surgeon are of great importance for the accurate identification of SLNs. Interestingly, a study [46] focused on the learning curve for the SLN mapping method concluded that at least 30 procedures are required to achieve a bilateral detection rate of greater than or equal to 75% [46]. In our study, all surgical procedures were performed at a single center by two highly experienced surgeons. The bilateral detection rate was 77% for laparoscopic and 100% for laparotomic procedures.

Accurate assessment of SLN status requires ultrastaging, which involves an increased number of serial sectioning levels with subsequent additional immunohistochemical (IHC) staining. This methodology enables the detection of micrometastases and isolated tumor cells (ITCs). Micrometastases are groups of tumor cells that are 0.2–2 mm in diameter or contain more than 200 cells. ITCs are defined as clusters up to 0.2 mm in diameter or up to 200 cells. The presence of a micrometastasis classifies the SLN as positively metastatic (pN1mi). However, the detection of ITCs does not alter the tumor stage and is designated as pN0(i+) [19].

The prognostic significance of ITCs (pN0(i+)) remains uncertain to date [47]. It is crucial to mention a clinical study conducted at the Memorial Sloan Kettering Cancer Center, where the ultrastaging protocol enabled the additional detection of micrometastases and ITCs in 4.5% of cases [48]. According to the internal study protocol, lymph nodes were sectioned at 50-micron intervals and stained with hematoxylin and eosin. In the absence of tumor cells in the inspected samples, IHC staining was performed using anti-cytokeratin (AE1:AE3) antibodies [48]. The authors emphasized that the additional detection of micrometastases significantly impacted subsequent treatment strategies and disease prognosis.

The results of the double-blind prospective clinical trial NCT01818739 addressed the impact of ultrastaging on long-term oncological outcomes [49]. According to the obtained data, although the methodology enabled the detection of micrometastases and ITCs, this patient cohort did not demonstrate worsened overall survival rates. The authors also noted that standard intraoperative frozen section analysis is a cost-effective and preferable method compared to ultrastaging. In our study, standard intraoperative frozen section analysis with hematoxylin and eosin staining was performed. In the laparoscopic surgery group, no SLN involvement was detected in any case, while in the laparotomic surgery group, one micrometastasis was identified.

The physicochemical properties of the ICG molecule in the human body are of paramount importance. In aqueous environments, ICG molecules can readily aggregate [50], while in blood or lymph, ICG binds to proteins, which can stabilize and maintain the distance between ICG molecules. The fluorescence intensity of ICG in vivo may vary depending on the concentration of proteins and lipoproteins, as well as individual patient factors [38]. Therefore, determining the optimal timing for the intraoperative detection of ICG in SLNs during NIR fluorescence mapping remains a relevant and significant objective.

The NIR fluorescence of ICG dissolved in albumin is most intense at concentrations of approximately 1–10 mg/L [51]. The concentration of the fluorescence agent can decrease by approximately 10- to 100-fold from the injection site to the lymph nodes [52] and may further diminish with increasing time after administration [53].

If a high concentration of the fluorescent agent is administered, as in the case of creating an “ICG depot,” nearby lymph nodes may not be detected until the ICG concentration is sufficiently diluted. Due to the significant spectral overlap between the excitation and emission spectra of ICG in the ~780 to ~820 nm range, ICG is susceptible to concentration quenching. Consequently, as demonstrated in Figure 7d, the fluorescence intensity at high ICG concentrations exhibits a non-linear dependence, which complicates the assessment of ICG concentration distribution in tissues. The reduction in fluorescence intensity observed at high ICG concentrations may be attributed to the formation of aggregates, which have a lower fluorescence quantum yield than ICG monomers [54], as well as to the concentration quenching effect [36]. Furthermore, ICG is unstable in saline solutions, which can lead to its degradation over time [55].

The primary advantage of NIR fluorescence imaging using ICG stems from the minimal absorption and autofluorescence of endogenous fluorophores and water in the 700–900 nm range. ICG fluorescence is most commonly excited within the 760–785 nm range, and the fluorescent signal is detected in the 820–840 nm range [56]. In contrast, our results from studying the absorption properties of ICG dissolved in human lymph demonstrate a peak absorption at approximately 804 nm (Figure 7a).

Consequently, the VENERA-green system utilizes an 800 nm excitation laser source, which provides significantly higher excitation efficiency for ICG within the lymph. Furthermore, the system records the fluorescence signal across a substantially broader spectral range of 830–1000 nm. This expanded detection window enables the detection of red-shifted fluorescence components, thereby ensuring more reliable detection of SLNs even at higher ICG concentrations, when the ICG fluorescence peak shifts to longer wavelengths.

During light irradiation, ICG molecules can undergo photobleaching, partly due to the chemical decomposition of molecules by singlet oxygen [57,58]. The accuracy of quantitative fluorescence measurements may be potentially affected by photobleaching, defined as irreversible photochemical degradation of ICG [59]. The kinetics of this process are highly dependent on the molecular environment and the availability of oxygen, which can lead to a decrease in NIR signal intensity [60]. However, in biological fluids such as lymph, the interaction between ICG and proteins (e.g., albumin) stabilizes the dye in its monomeric form, thereby reducing the photobleaching rate compared to aqueous solutions [61]. Although direct assessment of photobleaching was not performed in this study, several measures were taken to minimize its impact. Specifically, the spectral fluorescence measurements were conducted using a low-intensity laser (λ_exc_ = 650 nm, P_max_ = 10–12 mW) with an exposure time for spectral recording of only 20–100 ms per point. Furthermore, for the purpose of video imaging (λ_exc_ = 800 nm), the power density on the tissue surface was maintained at a low level of 20–30 mW/cm^2^, thus ensuring minimal fluorophore degradation during intraoperative imaging.

Although some studies report antitumor efficacy of ICG-mediated photodynamic therapy (PDT) upon NIR light irradiation [62,63], ICG is not considered an efficient photosensitizer, as its triplet quantum yield is extremely low and the precise mechanism of its cytotoxicity remains unclear [64]. However, ICG can be used to assess blood flow and lymph flow in the area of laser irradiation during tumor PDT. NIR fluorescence imaging of ICG requires the use of dedicated, highly sensitive systems comprising infrared excitation light sources, specialized filters, and cameras for detecting the NIR fluorescent signal [64]. This requirement limits the broad clinical application of ICG.

A critical parameter in ICG mapping of the lymphatic system is the timing of the SLN search initiation after tracer administration. Currently, data on the optimal time interval between ICG injection and the start of fluorescence SLN mapping are inconsistent, as the mapping time can vary significantly [65], partly due to the specifics of surgical procedures. It has been reported that the results of bilateral ICG-based SLN detection in CC are comparable between open surgery (laparotomy) and minimally invasive surgery (laparoscopy) [66]. In our study, ICG-based SLN detection was feasible within 10–15 min after cervical injection in 75% of cases. Furthermore, the fluorescence intensity of SLNs at 10–15 min post-injection was higher than that observed at 20–25 min (Figure 6). Other studies report performing ICG injection into the cervix 20–30 min before opening the pelvic retroperitoneal space [67].

The results of spectral-fluorescence studies on calibrated optical phantoms confirmed our clinical observations: the ICG concentration in SLNs detected within the 10–15 min interval was higher than in those visualized 20–25 min after ICG administration. This indicates rapid lymphatic drainage dynamics and confirms the existence of an optimal time window for detection. The utilization of the spectral shift in ICG fluorescence in relation to its concentration in lymph enabled the precise identification of the ICG concentration within the detected SLNs, a task that is notoriously challenging to achieve using standard fluorescence navigation techniques.

Thus, the results obtained in this work demonstrate that intraoperative ICG fluorescence imaging possesses high sensitivity and enables real-time SLN mapping. The ICG detection method shows high sensitivity to SLNs but lacks specificity for metastases. In our case, metastases were detected in only 2.8% (1/36) of the identified SLNs. A potential strategy to overcome this limitation could be the use of various ICG conjugates for the specific visualization of tumor cells [68] or the implementation of wide-field fluorescence lifetime imaging microscopy (FLIM) [69,70]. These approaches may, in the future, allow for the precise real-time discrimination of tumor cells during surgical procedures.

The research value of the study is that it describes for the first time the absorption and fluorescence properties of ICG in human lymph. We demonstrated that the fluorescence emission peak shift serves as a more reliable quantitative marker than absolute intensity, as it is significantly less susceptible to technical and instrumental artifacts. The utilization of calibrated human lymph-based phantoms enabled the correlation of intraoperative data with actual ICG concentrations in tissues. This work establishes a foundation for the development of advanced navigation systems capable of determining the degree of ICG saturation in lymph nodes, thereby minimizing the subjectivity of surgeon-dependent visual assessment.

## 5. Conclusions

The obtained results demonstrate the efficacy of NIR fluorescence mapping of iliac SLNs in EC and CC using interstitial ICG injection into the cervix 10–25 min before imaging. Fluorescence mapping of SLNs was performed during laparoscopic and laparotomic surgeries with λ_exc_ = 800 nm and ICG fluorescence detection at wavelengths above 830 nm. In most cases (75%, n = 15), SLNs were detected 10–15 min after the ICG administration into the cervical stroma.

Spectral fluorescence analysis of the excised SLN biopsy specimens with λ_exc_ = 650 nm revealed intense ICG fluorescence in the λ = 750–950 nm range, with peaks in the 830–850 nm wavelength region. For the first time, the absorption and fluorescent properties of ICG dissolved in human lymph were investigated. The ICG demonstrated a distinct absorption peak at 804 nm, accompanied by a short-wavelength “shoulder” near 740 nm and a fluorescence peak in the 750–950 nm range. A concentration-dependent redshift of the ICG fluorescence peak was recorded with increasing concentration from 2 to 40 mg/L. This phenomenon can be utilized for intraoperative monitoring of ICG accumulation dynamics and determination of its concentration distribution in tissues.

## 6. Patents

This study was conducted at the National Medical Research Center for Radiology (NMRC) of the Ministry of Health of the Russian Federation, based at the N.A. Lopatkin Scientific Research Institute of Urology and Interventional Radiology—a branch of the NMRC for Radiology of the Ministry of Health of Russia, within the Department of Gynecology with Chemotherapy.

The study included 20 female patients aged 33 to 70 years (57 ± 11.5 years) with morphologically confirmed EC and CC. The inclusion and exclusion criteria are presented in Table 3.

Patients who had received neoadjuvant chemotherapy or radiotherapy were excluded from the study to avoid potential interference with ICG lymphatic transport dynamics caused by therapy-induced fibrosis or lymphatic vessel destruction.

All patients underwent radical hysterectomy: type A for EC or type C1 for CC. The clinical parameters of all patients (n = 20), including morphological tumor type, TNM classification, and type of surgery, are presented in Table 4.

## Figures and Tables

**Table 1:** Histopathology results of fluorescence SLNs.

SLN Biopsy Status	Laparoscopy	Laparotomy
Patients with detected SLN	100% (17/17)	100% (3/3)
Metastasis	0/17	1/3
Sensitivity of bilateral SLN detection, %	77% (13/17)	100% (3/3)

Abbreviations: SLNs—Sentinel lymph nodes.

**Table 2:** Clinical parameters of the SLNs detection.

Case	Hysterectomy Type	Operation Time, min	Number of Detected SLNs
1	Radical hysterectomy A type, pelvic and para-aortic lymphadenectomy	220	2
2	Radical hysterectomy A type	120	2
3	Radical hysterectomy C1, pelvic and para-aortic lymphadenectomy	250	2
4	Radical hysterectomy A type, pelvic lymphadenectomy	260	2
5	Radical hysterectomy A type	120	1
6	Radical hysterectomy A type, pelvic lymphadenectomy	120	2
7	Radical hysterectomy A type	100	2
8	Radical hysterectomy A type	90	2
9	Radical hysterectomy A type	100	2
10	Radical hysterectomy A type	110	1
11	Radical hysterectomy A type	130	2
12	Radical hysterectomy A type, pelvic lymphadenectomy	230	2
13	Radical hysterectomy A type	240	2
14	Radical hysterectomy A type	70	1
15	Radical hysterectomy A type	120	1
16	Radical hysterectomy A type, pelvic lymphadenectomy	75	2
17	Radical hysterectomy A type, pelvic lymphadenectomy	220	2
18	Radical hysterectomy A type	170	2
19	Radical hysterectomy A type	80	2
20	Radical hysterectomy C1 type, pelvic lymphadenectomy	150	2

Abbreviations: SLNs—Sentinel lymph nodes.

**Table 3:** Criteria for inclusion and exclusion of patients from the study.

No.	Inclusion Criteria	Exclusion Criteria
1	Patients aged 18–70 years	Rare non endometrioid EC and rare subtypes of CC
2	Histologic subtype of endometrium cancer—endometrial, histologic types of CC—squamous and adenocarcinoma	Refusal of surgery and the proposed treatment plan
3	EC I stage CC IB1-IIA1 stages	Hypersensitivity to indocyanine green
4	Signed informed consent	Life expectancy less than 3 months
5	Patient is capable of making the decision	Status on the scale ECOG 3–4
6	Status on the scale ECOG ≤ 2	Neoadjuvant therapy
7	Absence of distant metastasis	Lymphostasis of the lower extremities
8		Renal failure

Abbreviations: EC—endometrial cancer; CC—cervical cancer; ECOG—Eastern cooperative oncology group.

**Table 4:** Clinical parameters of the patients.

Case	Age	Weight, kg	BMI	Clinical Diagnosis	Morphological Tumor Type	TNM Classification	Surgical Access
1	70	80	30	EC	endometrioid adenocarcinoma G3	cT1aN0M0	Laparotomy
2	66	82	29	EC	endometrioid adenocarcinoma G2	cT1aN0M0	Laparoscopy
3	60	60	22	CC	squamous cell carcinoma G2	cT2a1N0M0	Laparotomy
4	38	56	20	EC	endometrioid adenocarcinoma G2	cT1aN0M0	Laparoscopy
5	63	83	30	EC	endometrioid adenocarcinoma G2	cT1aN0M0	Laparoscopy
6	56	94	34	EC	endometrioid adenocarcinoma G3	cT1aN0M0	Laparoscopy
7	53	66	24	EC	endometrioid adenocarcinoma G1	cT1aN0M0	Laparoscopy
8	44	112	41	EC	endometrioid adenocarcinoma G1	cT1aN0M0	Laparoscopy
9	54	54	20	EC	endometrioid adenocarcinoma G3	cT1aN0M0	Laparoscopy
10	70	84	35	EC	endometrioid adenocarcinoma G1	cT1aN0M0	Laparoscopy
11	35	102	34	EC	endometrioid adenocarcinoma G1	cT1aN0M0	Laparoscopy
12	58	72	28	EC	endometrioid adenocarcinoma G2	cT1bN0M0	Laparoscopy
13	61	73	30	EC	endometrioid adenocarcinoma G2	cT1aN0M0	Laparoscopy
14	62	59	19	EC	endometrioid adenocarcinoma G2	cT1aN0M0	Laparoscopy
15	56	84	34	EC	endometrioid adenocarcinoma G2	cT1aN0M0	Laparoscopy
16	67	105	37	EC	endometrioid adenocarcinoma G2	cT1bN0M0	Laparoscopy
17	56	80	29	EC	endometrioid adenocarcinoma G1	cT1bN0M0	Laparoscopy
18	38	100	33	EC	endometrioid adenocarcinoma G1	cT1aN0M0	Laparoscopy
19	61	53	23	EC	endometrioid adenocarcinoma G2	cT1aN0M0	Laparoscopy
20	33	64	25	CC	cervical adenocarcinoma	cT2a1N0M0	Laparotomy

Abbreviations: EC—Endometrial cancer; CC—Cervical cancer; BMI–Body mass index.

## Data Availability

The data presented in this study are available on request from the corresponding author.

## Supplementary Materials

The following supporting information can be downloaded at: https://www.mdpi.com/article/10.3390/pharmaceutics18020211/s1, Video S1: The intraoperative NIR fluorescence imaging of SLN using ICG_1, Video S2: The intraoperative NIR fluorescence imaging of SLN using ICG_2.

## Abbreviations

SLNs	Sentinel lymph nodes
ICG	Indocyanine green
FGS	Fluorescence-guided surgery
NIR	Near-infrared
MB	Methylene blue
EC	Endometrial cancer
CC	Cervical cancer
FBS	Fetal bovine serum
FDA	Food and Drug Administration
EMA	European Medicines Agency
NCCN	National Comprehensive Cancer Network
ESGO	European Society of Gynaecological Oncology
ESTRO	European SocieTy for Radiotherapy and Oncology
ESP	European Society of Pathology
IHC	Immunohistochemical
ITCs	Isolated tumor cells
PDT	Photodynamic therapy
FLIM	Fluorescence lifetime imaging microscopy

## References

[1] Neagoe O.C., Ionica M., Mazilu O. (2018). The role of pelvic lymphocele in the development of early postoperative complications. Medicine.

[2] Rogovskaya T.T., Sidoruk A.A., Meshkova I.E., Nekrasova E.A., Ibragimov Z.N., Berlev I.V., Urmancheeva A.F. (2017). Lymphocysts after laparoscopic and open hysterectomy with pelvic lymphadenectomy for endometrial cancer. Probl. Oncol..

[3] Zhang J., Ju X., Feng Z., Zhang X., Li J. (2022). Progressive resistance exercise training to prevent lower-limb lymphedema after cervical cancer surgery: A feasibility study. Asia-Pac. J. Oncol. Nurs..

[4] Insalaco G., Incognito G.G., Genovese F., Gulino F.A., Rivoli L., Ciancio F., Valenti G., Incognito D., Carbone L., Palumbo M. (2024). Impact of obesity in the identification of the sentinel lymph node in endometrial cancer: A retrospective, monocentric study and literature review. Arch. Gynecol. Obstet..

[5] Sá R.d.S., Rodrigues R.F.V.A., Bugalho L.A., da Silva S.U., Nazário A.C.P. (2023). Evaluation of the efficacy of using indocyanine green associated with fluorescence in sentinel lymph node biopsy. PLoS ONE.

[6] Bounds M.C., Endean E.D. (2018). Treatment of postoperative high-volume lymphatic complications using isosulfan blue. J. Vasc. Surg. Venous Lymphat. Disord..

[7] Jankulovska A., Stojanoski S., Stojcevski S., Aluloski I., Jovanovic R., Kunovska S.K., Tanturovski M., Manevska N., Petrusevska G., Miladinova D. (2023). The evaluation of sentinel lymph node biopsy using radiocolloid in first stage endometrial cancer. Mol. Imaging Radionucl. Ther..

[8] Onishi T., Mihara K., Matsuda S., Sakamoto S., Kuwahata A., Sekino M., Kusakabe M., Handa H., Kitagawa Y. (2022). Application of magnetic nanoparticles for rapid detection and in situ diagnosis in clinical oncology. Cancers.

[9] Buda A., Crivellaro C., Elisei F., Di Martino G., Guerra L., De Ponti E., Cuzzocrea M., Giuliani D., Sina F., Magni S. (2016). Impact of indocyanine green for sentinel lymph node mapping in early stage endometrial and cervical cancer: Comparison with conventional radiotracer 99mTc and/or blue dye. Ann. Surg. Oncol..

[10] Baeten I.G.T., Hoogendam J.P., Jeremiasse B., Braat A.J.A.T., Veldhuis W.B., Jonges G.N., Jürgenliemk-Schulz I.M., van Gils C.H., Zweemer R.P., Gerestein C.G. (2022). Indocyanine green versus technetium-99m with blue dye for sentinel lymph node detection in early-stage cervical cancer: A systematic review and meta-analysis. Cancer Rep..

[11] van den Berg N.S., Brouwer O.R., Klop W.M.C., Karakullukcu B., Zuur C.L., Tan I.B., Balm A.J.M., van den Brekel M.W.M., Olmos R.A.V., van Leeuwen F.W.B. (2012). Concomitant radio- and fluorescence-guided sentinel lymph node biopsy in squamous cell carcinoma of the oral cavity using ICG-99mTc-nanocolloid. Eur. J. Nucl. Med..

[12] Wang L., Liu S., Xu T., Yuan L., Yang X. (2021). Sentinel lymph node mapping in early-stage cervical cancer: Meta-analysis. Medicine.

[13] Liu P., Tan J., Song Y., Huang K., Zhang Q., Xie H. (2022). The application of magnetic nanoparticles for sentinel lymph node detection in clinically node-negative breast cancer patients: A systemic review and meta-analysis. Cancers.

[14] Hameed S., Dai Z. (2018). Near-infrared fluorescence probes for surgical navigation. Mater. Today Chem..

[15] Zhang R.R., Schroeder A.B., Grudzinski J.J., Rosenthal E.L., Warram J.M., Pinchuk A.N., Eliceiri K.W., Kuo J.S., Weichert J.P. (2017). Beyond the margins: Real-time detection of cancer using targeted fluorophores. Nat. Rev. Clin. Oncol..

[16] Ministry of Health of the Russian Federation (2024). Clinical Guidelines: Cervical Cancer. https://cr.minzdrav.gov.ru/preview-cr/460_4.

[17] National Comprehensive Cancer Network (NCCN) (2025). NCCN Clinical Practice Guidelines in Oncology: Cervical Cancer. Version 1.2025. https://www.nccn.org/guidelines/guidelines-detail?category=1&id=1426.

[18] National Comprehensive Cancer Network (NCCN) (2025). NCCN Clinical Practice Guidelines in Oncology: Uterine Neoplasms. Version 1.2025. https://www.nccn.org/guidelines/guidelines-detail?category=1&id=1473.

[19] Concin N., Matias-Guiu X., Vergote I., Cibula D., Mirza M.R., Marnitz S., Ledermann J., Bosse T., Chargari C., Fagotti A. (2021). ESGO/ESTRO/ESP guidelines for the management of patients with endometrial carcinoma. Int. J. Gynecol. Cancer.

[20] Cibula D., Raspollini M.R., Planchamp F., Centeno C., Chargari C., Felix A., Fischerová D., Jahnn-Kuch D., Joly F., Kohler C. (2023). ESGO/ESTRO/ESP Guidelines for the management of patients with cervical cancer—Update 2023. Virchows Arch..

[21] Bogacz P., Pelc Z., Mlak R., Sędłak K., Kobiałka S., Mielniczek K., Leśniewska M., Chawrylak K., Polkowski W., Rawicz-Pruszyński K. (2025). Sentinel lymph node biopsy in breast cancer: The role of ICG fluorescence after neoadjuvant chemotherapy. Breast Cancer Res. Treat..

[22] Wölffer M., Liechti R., Constantinescu M., Lese I., Zubler C. (2024). Sentinel lymph node detection in cutaneous melanoma using indocyanine green-based near-infrared fluorescence imaging: A systematic review and meta-analysis. Cancers.

[23] Loverro M., Bizzarri N., Capomacchia F.M., Watrowski R., Querleu D., Gioè A., Naldini A., Santullo F., Foschi N., Fagotti A. (2024). Indocyanine green fluorescence applied to gynecologic oncology: Beyond sentinel lymph node. Int. J. Surg..

[24] Dai Z.-Y., Shen C., Mi X.-Q., Pu Q. (2023). The primary application of indocyanine green fluorescence imaging in surgical oncology. Front. Surg..

[25] Hoven P.V.D., Osterkamp J., Nerup N., Svendsen M.B.S., Vahrmeijer A., Van Der Vorst J.R., Achiam M.P. (2023). Quantitative perfusion assessment using indocyanine green during surgery—Current applications and recommendations for future use. Langenbeck’s Arch. Surg..

[26] Blanco-Colino R., Espin-Basany E. (2018). Intraoperative use of ICG fluorescence imaging to reduce the risk of anastomotic leakage in colorectal surgery: A systematic review and meta-analysis. Tech. Coloproctol..

[27] Nusrath S., Kalluru P., Shukla S., Dharanikota A., Basude M., Jonnada P., Abualjadayel M., Alabbad S., Mir T.A., Broering D.C. (2024). Current status of indocyanine green fluorescent angiography in assessing perfusion of gastric conduit and oesophago-gastric anastomosis. Int. J. Surg..

[28] Castagneto-Gissey L., Russo M.F., Iodice A., Casella-Mariolo J., Serao A., Picchetto A., D’ambrosio G., Urciuoli I., De Luca A., Salvati B. (2022). Intracholecystic versus intravenous indocyanine green (ICG) injection for biliary anatomy evaluation by fluorescent cholangiography during laparoscopic cholecystectomy: A case–control study. J. Clin. Med..

[29] Katsimperis S., Tzelves L., Bellos T., Manolitsis I., Mourmouris P., Kostakopoulos N., Pyrgidis N., Somani B., Papatsoris A., Skolarikos A. (2024). The use of indocyanine green in partial nephrectomy: A systematic review. Central Eur. J. Urol..

[30] Di Mitri M., Di Carmine A., Zen B., Collautti E., Bisanti C., D’antonio S., Libri M., Gargano T., Lima M. (2025). Advancing Pediatric Surgery with Indocyanine Green (ICG) Fluorescence Imaging: A Comprehensive Review. Children.

[31] He K., Li P., Zhang Z., Liu J., Liu P., Gong S., Chi C., Liu P., Chen C., Tian J. (2022). Intraoperative near-infrared fluorescence imaging can identify pelvic nerves in patients with cervical cancer in real time during radical hysterectomy. Eur. J. Nucl. Med..

[32] Capozzi V.A., Monfardini L., Sozzi G., Armano G., Rosati A., Alletti S.G., Cosentino F., Ercoli A., Cianci S., Berretta R. (2021). Subcutaneous vulvar flap viability evaluation with near-infrared probe and indocyanine green for vulvar cancer reconstructive surgery: A feasible technique. Front. Surg..

[33] Zammarrelli W.A., Afonso A.M., Broach V., Sonoda Y., Zivanovic O., Mueller J.J., Leitao M.M., Chan A., Abu-Rustum N.R. (2021). Sentinel lymph node biopsy in patients with endometrial cancer and an indocyanine green or iodinated contrast reaction—A proposed management algorithm. Gynecol. Oncol..

[34] Li X., Zhou M., Li S., Zhang F., Li Z., Li Z., Jin B. (2024). Solvent effects and self-assembled aggregation modulate nonlinear optical effects in indocyanine green-like dyes. Opt. Mater..

[35] Chon B., Ghann W., Uddin J., Anvari B., Kundra V. (2023). Indocyanine green (ICG) fluorescence is dependent on monomer with planar and twisted structures and inhibited by h-aggregation. Int. J. Mol. Sci..

[36] Cosco E.D., Lim I., Sletten E.M. (2021). Photophysical properties of indocyanine green in the shortwave infrared region. ChemPhotoChem.

[37] Mordon S., Devoisselle J.M., Soulie-Begu S., Desmettrea T. (1998). Indocyanine green: Physicochemical factors affecting its fluorescencein vivo. Microvasc. Res..

[38] Kraft J.C., Ho R.J.Y. (2014). Interactions of indocyanine green and lipid in enhancing near-infrared fluorescence properties: The basis for near-infrared imaging in vivo. Biochemistry.

[39] Kong S.-H., Noh Y.-W., Suh Y.-S., Park H.S., Lee H.-J., Kang K.W., Kim H.C., Lim Y.T., Yang H.-K. (2015). Evaluation of the novel near-infrared fluorescence tracers pullulan polymer nanogel and indocyanine green/γ-glutamic acid complex for sentinel lymph node navigation surgery in large animal models. Gastric Cancer.

[40] Efendiev K., Alekseeva P., Skobeltsin A., Shiryaev A., Pisareva T., Akhilgova F., Mamedova A., Reshetov I., Loschenov V. (2024). Combined use of 5-ALA-induced protoporphyrin IX and chlorin e6 for fluorescence diagnostics and photodynamic therapy of skin tumors. Lasers Med. Sci..

[41] Dinoi G., Ghoniem K., Huang Y., Zanfagnin V., Cucinella G., Langstraat C., Glaser G., Kumar A., Weaver A., McGree M. (2024). Endometrial cancer with positive sentinel lymph nodes: Pathologic characteristics of metastases as predictors of extent of lymphatic dissemination and prognosis. Int. J. Gynecol. Cancer.

[42] Lee E.-H., Kim J.-K., Lim J.-S., Lim S.-J. (2015). Enhancement of indocyanine green stability and cellular uptake by incorporating cationic lipid into indocyanine green-loaded nanoemulsions. Colloids Surf. B Biointerfaces.

[43] Berlev I., Trifanov Y., Kanaev S., Urmancheeva A., Mikaya N., Bezhanova Y., Nekrasova Y., Mkrtchyan G., Rogovskaya T., Krzhivitskiy P. (2017). Possibilities of detection of sentinel lymph nodes in endometrial cancer by radioisotope and fluorescent (icg) methods. Probl. Oncol..

[44] Frumovitz M., Plante M., Lee P.S., Sandadi S., Lilja J.F., Escobar P.F., Gien L.T., Urbauer D.L., Abu-Rustum N.R. (2018). A randomized phase III multicenter study assessing near infrared fluorescence in the detection of sentinel lymph nodes in women with cervical and uterine cancers: The FILM Trial. Lancet Oncol..

[45] Maramai M., Achilarre M., Aloisi A., Betella I., Bogliolo S., Garbi A., Maruccio M., Quatrale C., Aletti G., Mariani A. (2021). Cervical re-injection of indocyanine green to improve sentinel lymph node detection in endometrial cancer. Gynecol. Oncol..

[46] Murase R., Tanaka H., Hamakawa T., Goda H., Tano T., Ishikawa A., Hino S., Sumida T., Nakashiro K., Hamakawa H. (2015). Double sentinel lymph node mapping with indocyanine green and 99m-technetium–tin colloid in oral squamous cell carcinoma. Int. J. Oral Maxillofac. Surg..

[47] Holloway R.W., Abu-Rustum N.R., Backes F.J., Boggess J.F., Gotlieb W.H., Lowery W.J., Rossi E.C., Tanner E.J., Wolsky R.J. (2017). Sentinel lymph node mapping and staging in endometrial cancer: A Society of Gynecologic Oncology literature review with consensus recommendations. Gynecol. Oncol..

[48] Kim C.H., Soslow R.A., Park K.J., Barber E.L., Khoury-Collado F., Barlin J.N., Sonoda Y., Hensley M.L., Barakat R.R., Abu-Rustum N.R. (2013). Pathologic ultrastaging improves micrometastasis detection in sentinel lymph nodes during endometrial cancer staging. Int. J. Gynecol. Cancer.

[49] Backes F.J., Cohen D., Salani R., Cohn D.E., O’Malley D.M., Fanning E., Suarez A.A., Fowler J.M. (2019). Prospective clinical trial of robotic sentinel lymph node assessment with isosulfane blue (ISB) and indocyanine green (ICG) in endometrial cancer and the impact of ultrastaging (NCT01818739). Gynecol. Oncol..

[50] Saxena V., Sadoqi M., Shao J. (2003). Degradation kinetics of indocyanine green in aqueous solution. J. Pharm. Sci..

[51] Kong S.-H., Bae S.-W., Suh Y.-S., Lee H.-J., Yang H.-K. (2018). Near-infrared fluorescence lymph node navigation using indocyanine green for gastric cancer surgery. J. Minim. Invasive Surg..

[52] Li C., Xu X., McMahon N., Ibrahim O.A., Sattar H.A., Tichauer K.M. (2019). Paired-agent fluorescence molecular imaging of sentinel lymph nodes using indocyanine green as a control agent for antibody-based targeted agents. Contrast Media Mol. Imaging.

[53] Proulx S.T., Luciani P., Derzsi S., Rinderknecht M., Mumprecht V., Leroux J.-C., Detmar M. (2010). Quantitative imaging of lymphatic function with liposomal indocyanine green. Cancer Res..

[54] Maarek J.M.I., Holschneider D.P., Harimoto J. (2001). Fluorescence of indocyanine green in blood: Intensity dependence on concentration and stabilization with sodium polyaspartate. J. Photochem. Photobiol. B Biol..

[55] Alotaibi H., Hatahet T., Al-Jamal W.T. (2024). Indocyanine green J-aggregate (IJA) theranostics: Challenges and opportunities. Int. J. Pharm..

[56] Marshall M.V., Rasmussen J.C., Tan I.-C., Aldrich M.B., Adams K.E., Wang X., Fife C.E., Maus E.A., Smith L.A., Sevick-Muraca E.M. (2010). Near-infrared fluorescence imaging in humans with indocyanine green: A review and update. Open Surg. Oncol. J..

[57] Engel E., Schraml R., Maisch T., Kobuch K., König B., Szeimies R.M., Hillenkamp J., Bäumler W., Vasold R. (2008). Light-induced decomposition of indocyanine green. Investig. Ophthalmol. Vis. Sci..

[58] Alekseeva P., Makarov V., Efendiev K., Shiryaev A., Reshetov I., Loschenov V. (2024). Devices and methods for dosimetry of personalized photodynamic therapy of tumors: A review on recent trends. Cancers.

[59] Alekseeva P., Makarov V., Efendiev K., Gilyadova A., Loschenov V. (2025). Advances in photodynamic treatment of precancerous and cancerous gynecological diseases. Cancers.

[60] Clutter E.D., Chen L.L., Wang R.R. (2022). Role of photobleaching process of indocyanine green for killing neuroblastoma cells. Biochem. Biophys. Res. Commun..

[61] Tovar J.S.D., Kassab G., Inada N.M., Bagnato V.S., Kurachi C. (2023). Photobleaching kinetics and effect of solvent in the photophysical properties of indocyanine green for photodynamic therapy. ChemPhysChem.

[62] Papayan G., Akopov A. (2018). Potential of indocyanine green near-infrared fluorescence imaging in experimental and clinical practice. Photodiagn. Photodyn. Ther..

[63] Houthoofd S., Vuylsteke M., Mordon S., Fourneau I. (2020). Photodynamic therapy for atherosclerosis. The potential of indocyanine green. Photodiagn. Photodyn. Ther..

[64] Dsouza A.V., Lin H., Henderson E.R., Samkoe K.S., Pogue B.W. (2016). Review of fluorescence guided surgery systems: Identification of key performance capabilities beyond indocyanine green imaging. J. Biomed. Opt..

[65] Rocha A., Domínguez A.M., Lécuru F., Bourdel N. (2016). Indocyanine green and infrared fluorescence in detection of sentinel lymph nodes in endometrial and cervical cancer staging—A systematic review. Eur. J. Obstet. Gynecol. Reprod. Biol..

[66] Bizzarri N., Luigi P.A., Ferrandina G., Zannoni G.F., Carbone M.V., Fedele C., Teodorico E., Gallotta V., Alletti S.G., Chiantera V. (2021). Sentinel lymph node mapping with indocyanine green in cervical cancer patients undergoing open radical hysterectomy: A single-institution series. J. Cancer Res. Clin. Oncol..

[67] Muallem M.Z., Sayasneh A., Armbrust R., Sehouli J., Miranda A. (2021). Sentinel lymph node staging with indocyanine green for patients with cervical cancer: The safety and feasibility of open approach using SPY-PHI technique. J. Clin. Med..

[68] Hu D., Zha M., Zheng H., Gao D., Sheng Z. (2025). Recent advances in indocyanine green-based probes for second near-infrared fluorescence imaging and therapy. Research.

[69] Pal R., Lwin T.M., Krishnamoorthy M., Collins H.R., Chan C.D., Prilutskiy A., Nasrallah M.P., Dijkhuis T.H., Shukla S., Kendall A.L. (2023). Fluorescence lifetime of injected indocyanine green as a universal marker of solid tumours in patients. Nat. Biomed. Eng..

[70] Krishnamoorthy M., Pal R., Hou S.S., Choi H.S., Bacskai B.J., Bogdanov A.A., Tanabe K.K., Varvares M.A., Kumar A.T.N. (2025). High-speed wide-field fluorescence lifetime imaging for intraoperative tumor visualization and in vivo multiplexing. Biomed. Opt. Express.

